# Embodying an outgroup: the role of racial bias and the effect of multisensory processing in somatosensory remapping

**DOI:** 10.3389/fnbeh.2013.00165

**Published:** 2013-11-18

**Authors:** Chiara Fini, Flavia Cardini, Ana Tajadura-Jiménez, Andrea Serino, Manos Tsakiris

**Affiliations:** ^1^Department of Neuroscience and Imaging, University G. d'Annunzio, and ITAB, Institute for Advanced Biomedical Technologies, G. d'Annunzio FoundationChieti, Italy; ^2^Lab of Action and Body, Department of Psychology, Royal Holloway, University of LondonEgham, UK; ^3^Department of Computer Science, UCL Interaction Center, University College LondonLondon, UK; ^4^Laboratory of Cognitive Neuroscience and Center for Neuroprosthetics, School of Life Sciences, École Polytechnique Fédérale de LausanneÉcublens, Switzerland; ^5^Department of Psychology, Alma Mater Studiorum, University of BolognaBologna, Italy

**Keywords:** multisensory interaction, visual remapping of touch, interpersonal multisensory stimulation, implicit racial bias, enfacement illusion

## Abstract

We come to understand other people's physical and mental states by re-mapping their bodily states onto our sensorimotor system. This process, also called somatosensory resonance, is an essential ability for social cognition and is stronger when observing ingroup than outgroup members. Here we investigated, first, whether implicit racial bias constrains somatosensory resonance, and second, whether increasing the ingroup/outgroup perceived physical similarity results in an increase in the somatosensory resonance for outgroup members. We used the Visual Remapping of Touch effect as an index of individuals' ability in resonating with the others, and the Implicit Association Test to measure racial bias. In Experiment 1, participants were asked to detect near-threshold tactile stimuli delivered to their own face while viewing either an ingroup or an outgroup face receiving a similar stimulation. Our results showed that individuals' tactile accuracy when viewing an outgroup face being touched was negatively correlated to their implicit racial bias. In Experiment 2, participants received the interpersonal multisensory stimulation (IMS) while observing an outgroup member. IMS has been found to increase the perceived physical similarity between the observer's and the observed body. We tested whether such increase in ingroup/outgroup perceived physical similarity increased the remapping ability for outgroup members. We found that after sharing IMS experience with an outgroup member, tactile accuracy when viewing touch on outgroup faces increased. Interestingly, participants with stronger implicit bias against the outgroup showed larger positive change in the remapping. We conclude that shared multisensory experiences might represent one key way to improve our ability to resonate with others by overcoming the boundaries between ingroup and outgroup categories.

## Introduction

A conscious experience of one's body as one's own is an essential feature of human experience (Gallup and Suarez, 1986; Graziano and Cooke, 2006). An important mental construct that ultimately contributes to this experience by attributing sensations, events and objects either to one's self or to the others, is self-identification—i.e., the cognitive capacity for recognizing one's body as belonging to oneself and as distinct from other individuals (Bermudez et al., 1995; Aglioti and Candidi, 2011). Importantly, a crucial distinction that has been recently highlighted relates to the neural mechanisms automatically recruited when perceiving our self or the others. Cardini and colleagues recently suggested that whereas the brain constantly tries to maintain *multisensory* coherence in representing one's own body—by continuously integrating visual, tactile and proprioceptive inputs—recognition of others' bodies usually occurs through *unisensory* inputs that drive pure visual representations of the others (Cardini et al., 2013). The contribution of multisensory integration to self-awareness has been well documented across several bodily illusions that use synchronous visual and tactile input to induce a sense of body ownership over a foreign body-part (see Rubber Hand Illusion; Botvinick and Cohen, 1998), a whole body (see the full body illusion; Lenggenhager et al., 2007), and a face (see the enfacement illusion, Tsakiris, 2008). However, accumulating evidence has recently suggested a multisensory basis of social cognition, by demonstrating the ability of our brain to recruit the neural structures involved in processing one's motor (Rizzolatti et al., 2001), sensory (Ishida et al., 2010) and affective states (Adolphs et al., 2000; Carr et al., 2003) also when such states are merely *observed* in others. Importantly, our ability to access to others' experiences strongly depends on the automaticity of this *simulation* mechanism (Gallese et al., 2004; Bernhardt and Singer, 2012).

However, this simulation process might be constrained by social factors such as prejudice, stereotypes and discrimination bias. For instance, when participants observe others experiencing pain, they show a modulation of corticospinal excitability that is thought to reflect sensorimotor resonance with others (Avenanti et al., 2005). However, when the painful stimulus is delivered to a person belonging to an outgroup, sensorimotor resonance vanishes (Avenanti et al., 2010; Gutsell and Inzlicht, 2010, 2012). Importantly, the lack of sensorimotor resonance with outgroup members correlates with participants' implicit racial bias against that outgroup, as measured by the Implicit Association Task (IAT) (Greenwald et al., 1998). Interestingly, such relationship might be bidirectional. Mathur and colleagues found that extraordinary empathy for ingroup members was associated with enhanced activity in the medial prefrontal cortex, which in turn was predictive of altruistic motivation and prosocial behavior toward members of one's own ethnic group (Mathur et al., 2010). These studies aptly highlight the complex interactions between social cognition and bodily remapping. However, they do not directly consider the role of self-representations in social interactions and especially its potential malleability, which might be a critical factor for understanding not only the differences in how we resonate with the others' physical and affective states, but also the ways in which such differences can be altered. Importantly in a recent study, Maister and colleagues found that experiencing a sense of body ownership over a hand of different color in turn affects implicit racial bias (Maister et al., 2013). Taken together these results highlight a rather complex relationship between automatic and relatively low-level sensorimotor resonance mechanisms between self and others and higher level representation of the others in relationship to the self. Here we first studied the relationship between somatosensory resonance and racial biases by investigating to which extent implicit racial biases predict sensory resonance between self and other. Second, whether blurring the self-other boundaries, by means of multisensory-induced changes in self-face representation, might change somatosensory resonance between individuals belonging to different ethnic groups.

In Experiment 1, we took advantage of the Visual Remapping of Touch effect (VRT) (Ladavas and Serino, 2010) to quantify somatosensory resonance between self and other. Viewing a face being touched by fingers enhances the perception of near-threshold tactile stimuli on the face as compared to viewing the same face being just approached (Serino et al., 2008; Cardini et al., 2011). The VRT effect is body-specific (i.e., for viewing touch on body-parts), is higher when observing one's own face as compared to when observing the face of another person (Serino et al., 2008), and is modulated by social factors, i.e., it is stronger when touch is viewed on the face of another person belonging to the same ethnic, or even political, group as oneself, as compared to when touch is viewed on the face of an outgroup member (Serino et al., 2009). In addition, in this experiment we used the IAT (Greenwald et al., 1998) to quantify individuals' implicit prejudice toward outgroup members, and the Blatant and Subtle Prejudice Scale by Pettigrew and Merteens (Pettigrew and Meertens, 1995) and a social-political opinions scale (Manganelli Rattazzi and Volpato, 2001) to assess more explicit forms of racial prejudice. We hypothesized that the lack of VRT effect for outgroup members (Serino et al., 2009) could be modulated by pre-existing individual differences in racial bias, whereby lower levels of racial bias would predict a stronger VRT effect for outgroup faces.

In Experiment 2, we tested the hypothesis that reduced somatosensory resonance for outgroup others can be overcome if participants share a multisensory experience with the other that results in a blurring of the self-other boundaries. In the so called “Enfacement Illusion,” an interpersonal multisensory stimulation (IMS) consisting in seeing the face of someone else being touched, while stimulation on one's own face, induces a sense of identification with the viewed person and increases the perceived physical similarity between the self and the other's face (Tsakiris, 2008; Sforza et al., 2010; Tajadura-Jiménez et al., 2012a). We have recently found that after inducing the enfacement illusion by IMS the remapping between somatosensory stimuli felt on one's body and seen on the other's body is enhanced so that there is no more difference between VRT effect for the self and the other's face (Cardini et al., 2012b), suggesting that increasing the perceived similarity between self and other (Tajadura-Jiménez et al., 2012b) enhances somatosensory resonance between self and others.

In line with our previous finding, here, we similarly hypothesized that by inducing the enfacement illusion through IMS for outgroup faces might reduce the ingroup/outgroup perceived physical difference and eventually reduce the ingroup/outgroup bias previously found in the VRT effect (Serino et al., 2009). Therefore, VRT was measured before and after the enfacement illusion. Higher VRT effect for outgroup faces was expected after participants have experienced the enfacement illusion. Finally, as individual differences in the racial bias might modulate the strength of the VRT for outgroup faces, as tested in Experiment 1, we also considered whether implicit racial biases might affect the malleability of the VRT effect through the enfacement illusion.

## Experiment 1

### Materials and methods

#### Participants

Twenty-four Caucasian volunteers (*M*_age_ 24.6 years, 21 females, all but one right-handed, all with normal or corrected-to-normal vision and reported normal touch) from the University of Cesena consented to participate in this study, approved by the Ethical committee of the Psychology Department, University of Bologna.

#### Stimuli preparation

Prior to the experiment, videos depicting a model's face being touched or just approached bilaterally or unilaterally by human fingers were recorded. Two female and two male models were used. For each gender, one model was of White and one of Black ethnic origin, and they matched for trustworthiness and attractiveness. A total of 24 videos were produced.

#### Design

We employed a 2 × 2 Factorial design, adapted from Serino et al. (2009), where the first factor was the Face that participants saw, i.e., an Ingroup (White) or Outgroup (Black) face, and the second was the Fingers' Trajectory, i.e., two fingers touching or no-touching the seen face. Unilateral visual and tactile stimulations were used as catch trials, hence not considered experimental factors (see below).

#### Procedure

First, participants completed the two-category (White vs. Black faces) race IAT that measures implicit racial attitudes (Greenwald et al., 1998). Next, we administered the Pettigrew and Merteens' subtle and blatant subscales of prejudice (Pettigrew and Meertens, 1995) to measure participants' explicit racial attitudes and asked participants to complete a social-political opinions scale aimed at measuring individual tendencies to apply a restricted policy toward immigrants (Manganelli Rattazzi and Volpato, 2001).

Next, participants performed an experimental session where the VRT effect was measured when viewing touch toward an Ingroup or an Outgroup face. Tactile stimuli were delivered by two constant current electrical stimulators (DS7A, Digitimer), via two couples of surface electrodes placed on the participants' cheeks.

**Figure 1:**
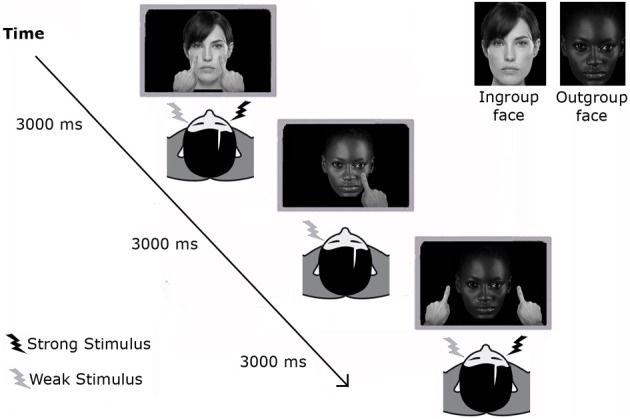
**Experimental paradigm in Experiment 1.** Participants performed three randomized blocks of tactile confrontation task, lasting ~3 min each. In each trial a different image (either an Ingroup or an Outgroup face) was presented in the video, where fingers moved toward the image and then backwards to their starting position. Fingers either touched the cheeks of the shown face or stopped 5 cm alongside the face. As soon as the fingers reached the image, a tactile input was delivered on the participants' cheeks. Participants were asked to indicate the side on their face in which they *felt* the tactile stimulation, regardless of visual stimulation.

Before the experimental task, the tactile stimulus on one cheek was set to be more intense (threshold detection rate of ≈100%) than that on the other cheek (≈60%) through a staircase procedure as used in previous studies (for a full description of the procedure, see Cardini et al., 2012a). Participants were asked to watch pre-recorded videos on a monitor placed ≈60 cm in front of them. The videos depicted a face that was, in different randomized trials, the Ingroup or the Outgroup face, matched for the participant's gender. The videos showed one or two fingers moving toward the image of the face and then backwards to their starting position. In different trials the fingers touched the cheeks of the face (Touch), or stopped about 5 cm beside it (No-Touch) (Figure 1). Visual stimuli approaching or touching the observed face and tactile stimuli delivered to the participant's face were simultaneous so that when the fingers reached the end point of the forward trajectory a tactile stimulation (the unilateral weak, the unilateral strong or both stimuli) was delivered to the participant's face. Participants were asked to indicate by unspeeded key-presses the side on their face in which they *felt* the tactile stimulation, regardless of visual stimulation. Three different buttons were used to report the three possible sites of the face (“D” button for the left cheek, “K” button for the right cheek, or “SPACE” bar for both cheeks). A PC running C.I.R.O. software was used to control the presentation of the stimuli and record responses. At the beginning of each block, detection thresholds were recalibrated to ensure comparable threshold detection rate.

Stimuli comprised a combination of the two different Faces (Ingroup and Outgroup), two types of tactile stimulation (Unilateral and Bilateral), two types of visual stimulation (Unilateral and Bilateral), and two fingers' trajectories (Touch and No-Touch). To maximize the number of critical trials and ensure the unpredictable nature of the task, stimuli were presented with different frequencies and only some of these combinations were used as experimental trials, whereas the other ones were used as catch trials. In particular, the combinations of the two images being touched, or just approached bilaterally while participants received a bilateral tactile stimulation were repeated 12 times each (*bilateral* conditions); all the other combinations using either unilateral visual, unilateral tactile or unilateral visual and tactile stimulation were used as catch trials, thus repeated only few times each (*unilateral* conditions). 136 trials in total were presented across three blocks. Accuracy scores were computed for each Face and in each *bilateral* condition (Touch and No-Touch) separately, by measuring the percentage of correctly reported bilateral touches delivered on the participant's face, while viewing the Ingroup face being touched (Ingroup_Touch condition), the Outgroup face being touched (Outgroup_Touch condition), the Ingroup face being just approached (Ingroup_No-Touch condition) or the Outgroup face being just approached (Outgroup_No-Touch condition). In addition, for Ingroup and Outgroup faces separately, we computed an index of the VRT effect as the difference in accuracy in detecting bilateral tactile stimulation in the Touch and No-Touch fingers' trajectory conditions (VRT_Ingroup: Ingroup_Touch minus Ingroup_No-Touch; VRT_Outgroup: Outgroup_Touch minus Outgroup_No-Touch) (see Table 1).

**Table 1:** **Mean scores (±standard error of the means indicated in brackets in italic font) for each Face (Ingroup and Outgroup) and for each Fingers' Trajectory (Touch and No-Touch) conditions**.

		**Mean accuracy (SD)**	**VRT effect (Touch-NoTouch)**
Ingroup	Touch	83% (3%)	5% (3%)
	No-Touch	78% (3%)	
Outgroup	Touch	80% (4%)	−2% (4%)
	No-Touch	82% (3%)	

The last column on the right shows the VRT effects calculated as the difference between the mean scores in the Touch and in the No-Touch conditions, for each face separately.

### Results

To investigate whether racial differences in the VRT effect, i.e., higher VRT effect when viewing ingroup as opposed to outgroup faces (Serino et al., 2009), are related to individuals' implicit racial bias, we ran an ANCOVA on the percentages of correct responses to *bilateral* tactile stimulation delivered on the participant's cheeks with the within-subjects factors of Face (Ingroup and Outgroup) and of Fingers' trajectory (Touch and No-Touch) and IAT scores as covariate. Conditions of *unilateral* visual and tactile stimulations were used as catch trials and hence not included in statistical analysis (as in Cardini et al., 2012b).

**Figure 2:**
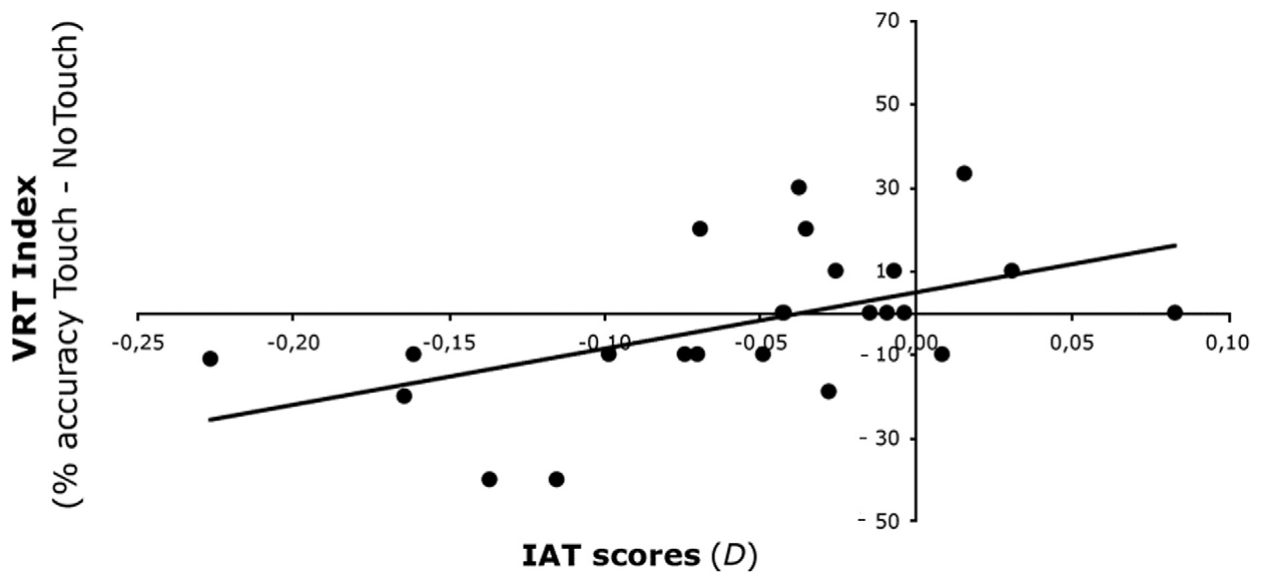
**Experiment 1 results.** Relationship between VRT effect for Outgroup faces and IAT scores. IAT scores (on the *x*-axis) predicted the VRT effect (on the *y*-axis)—expressed as the difference in detection of bilateral tactile stimulation in the Touch and No-Touch fingers' trajectory conditions.

The interaction Face X Fingers trajectory X IAT scores was significant [*F*_(1, 22)_ = 7.84, *p* < 0.05]. To interpret this 3-way interaction, we calculated an index of the VRT effect as the difference in detection of bilateral tactile stimulation in the Touch and No-touch fingers' trajectory conditions for Ingroup (Touch, *M* = 83%, s.e.m. = 3%; No-Touch, *M* = 78%, s.e.m. = 3%) and Outgroup faces (Touch, *M* = 80%, s.e.m. = 4%; No-Touch, *M* = 82%, s.e.m. = 3%). A positive correlation [*r* = 0.516, *p* < 0.05] was found between IAT scores and the VRT effect for the Outgroup face: the higher the IAT score—i.e., the more positive the attitude toward Black faces—the stronger the ability to remap touch seen on a Black face onto one's own face. Conversely, no significant correlation was found between IAT score and the VRT effect for the Ingroup face [*r* = −0.260, *p* = 0.22]. These results show that the previously observed lack of a VRT effect for outgroup faces (Serino et al., 2009) is related to individual implicit racial attitudes (Figure 2).

A multiple linear regression analysis was performed to test whether the VRT effect for the outgroup face was better predicted from individual scores obtained from the IAT, the Pettigrew and Merteens' subtle and blatant prejudice subscales or the social-political opinions scale. The overall model fit was significant, [*F*_(4, 23)_ = 3.44, *p* = 0.028], but only IAT scores positively predicted the VRT effect for the outgroup faces [β = 0.397, *t*_(19)_ = 2.114, *p* = 0.048], (for all other scales *p* > 0.05).

Taken together, the results of Experiment 1 replicate the racial modulation of the VRT effect (Serino et al., 2009) and extend previous results by showing that this effect depends on pre-existing individual differences on implicit, but not explicit, racial biases: individual implicit attitudes toward outgroup—assessed at the beginning of the experiment by the widely-used IAT—seem to modulate the strength with which we remap seen touch toward an outgroup face, onto the felt touch. The ease with which people tent to remap onto one's body sensory event observed on someone else's body gradually increases as the individual level of racial bias decreases. In the light of these results, in Experiment 2 we used the VRT effect as a measure of automatic, low-level racial bias in sensorimotor resonance and we tested whether we could act upon such bias by blurring the boundaries between the representation of one self and that of outgroup others. To that end, we used the “Enfacement Illusion” that has been shown to produce changes in identification and perceived self-other similarity (Tajadura-Jiménez et al., 2012b). We measured the VRT effect before and after participants saw an outgroup face being touched in synchrony or not with their own face. As before, IAT measures were included because we predicted that the effect of synchronous IMS might be modulated by individual differences in pre-existing implicit biases.

## Experiment 2

### Materials and methods

#### Participants

Thirty Caucasian volunteers (*M*_age_ 20.03 years, all females, right-handed, all with normal or corrected-to-normal vision and reported normal touch) from Royal Holloway, University of London consented to participate in this study, approved by the Ethical committee of the Psychology Department, Royal Holloway, University of London.

#### Stimuli preparation

Prior to the experiment, videos depicting a model's face being touched or just approached bilaterally or unilaterally by human fingers were recorded. Eight female models were used. Four models were of White (Ingroup) and four of Black (Outgroup) ethnic origin, and they matched for trustworthiness and attractiveness. A total of 48 videos were produced.

For the IMS session we recorded for each of the models a 2-min video depicting her face being touched on the right cheek by a cotton bud (every 3 s).

#### Design

**Figure 3:**
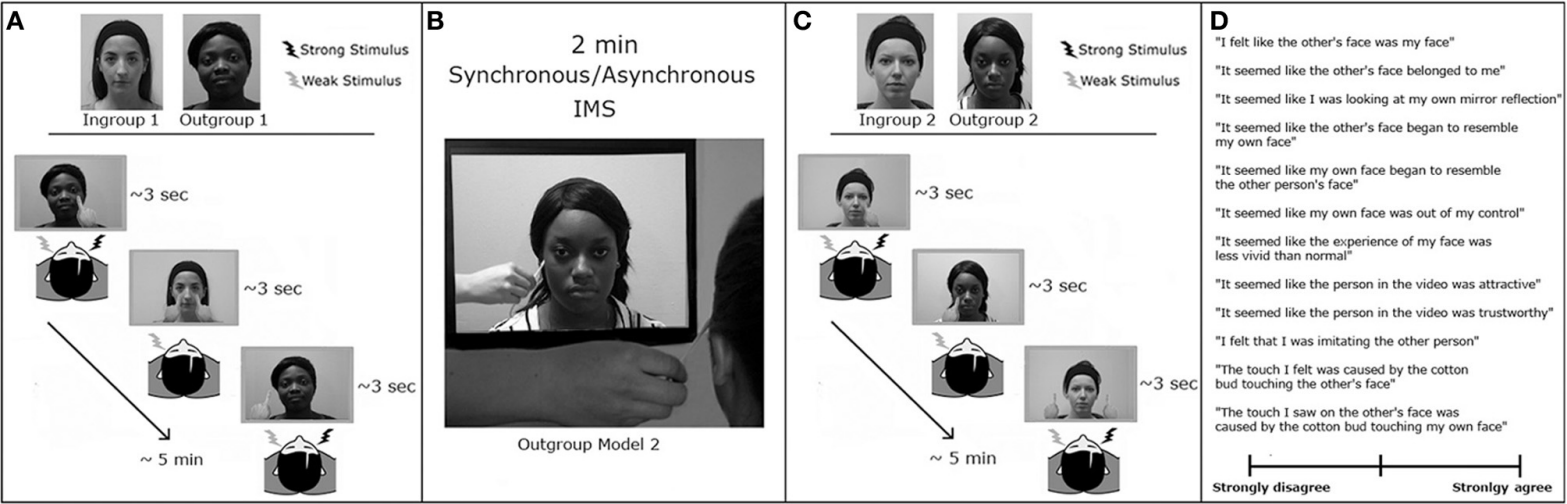
**Experimental paradigm in Experiment 2.** The experimental design comprised two experimental sessions, each comprising four consecutive blocks: **(A)** VRT measurement pre-IMS. This block was organized as in Experiment 1. **(B)** Interpersonal multisensory stimulation. For 2 min, participants were touched by a cotton bud on the left cheek every 3 s while watching a video showing an Outgroup face being touched with a cotton bud on a specularly congruent location in synchrony (in one session) or asynchrony (in the other session) with respect to the touch delivered on the participants' face. **(C)** VRT measurement post-IMS. This session was similar to the one before IMS, but now the Outgroup face was the face seen during the IMS. **(D)** Participants were asked to rate their agreement with 12 statements about their experience during IMS, using a Visual Analog Scale.

The 2 × 2 × 2 × 2 Factorial design of the present experiment was adapted from Cardini et al. (2012b). The first factor was the Face that participants saw during the VRT session (Ingroup and Outgroup). The second factor was the Fingers' Trajectory (Touch and No-Touch). The third factor was the timing of the VRT task (pre- and post-IMS). The fourth factor was the type of IMS (synchronous and asynchronous visuo-tactile stimulation between the face of the model and the face of the participant). To independently assess whether participants experienced the Enfacement Illusion, after the completion of each post-IMS VRT session (Figure 3), participants were asked to rate their level of agreement with a set of 12 statements (adapted from Tajadura-Jiménez et al. (2012b) related to their subjective experience of identification with and ownership over the other's face, mirror-like exposure, feelings of control over the other's face, and affect toward the other's person during the IMS session (see Table 2).

**Table 2:** **Mean scores (± SD values indicated in italic font) for each of the 12 statements presented after both the synchronous and asynchronous interpersonal multisensory stimulation (IMS)**.

**Statements**	**Synch**	**Asynch**	***p***
*Q1: “I felt like the other's face was my face”*	−0.22 (1.63)	−1.38 (1.30)	0.000
*Q2: “It seemed like the other's face belonged to me”*	−0.62 (1.55)	−1.39 (1.36)	0.007
*Q3: “It seemed like I was looking at my own mirror reflection”*	−0.06 (1.58)	−1.35 (1.44)	0.000
*Q4: “It seemed like the other's face began to resemble my own face”*	−0.46 (1.56)	−1.25 (1.17)	0.001
*Q5: “It seemed like my own face began to resemble the other person's face”*	−0.65 (1.78)	−1.37 (1.21)	0.014
*Q6: “It seemed like my own face was out of my control”*	−0.03 (1.55)	−0.55 (1.27)	0.134
*Q7: “It seemed like the experience of my face was less vivid than normal”*	0.23 (1.07)	−0.07 (1.15)	0.159
*Q8: “It seemed like the person in the video was attractive”*	0.45 (0.82)	0.45 (1.10)	0.840
*Q9: “It seemed like the person in the video was trustworthy”*	0.74 (0.91)	0.76 (0.81)	0.708
*Q10: “I felt that I was imitating the other person”*	0.38 (1.54)	0.22 (1.50)	0.648
*Q11: “The touch I felt was caused by the cotton bud touching the other's face”*	−0.40 (1.50)	−1.24 (1.38)	0.001
*Q12: “The touch I saw on the other's face was caused by the cotton bud touching my own face”*	0.08 (1.57)	−1.64 (1.32)	0.000

Participants had to agree or disagree with each of the statements using a Visual Analog Scale (from −3, strongly disagree to +3, strongly agree). Some of the factors did not pass the normality test, therefore we used non-parametrical statistical tests to analyse the data. Wilcoxon Signed Ranks Test compared the answers to each of the statements after the synchronous and asynchronous IMS.

#### Procedure

First, we administered the race IAT. Next participants performed two experimental sessions, each consisting of a pre-IMS VRT block, an IMS block, a post-IMS VRT block, followed by the IMS questionnaire. The VRT was identical to that described for Experiment 1, with the only difference that a total of 100 trials were presented randomly ordered within one unique block, lasting ~5 min. A PC running NI LabVIEW 2011 software was used to present the stimuli and record responses. The difference between the two experimental sessions in Experiment 2 was the type of IMS (synchronous or asynchronous). The presentation order of the two experimental sessions was counter-balanced between participants. Each IMS block lasted 2 min, during which participants were touched by a cotton bud on the cheek while watching a pre-recorded video showing a face of an Outgroup model (i.e., Outgroup Model 2, that was different from that shown in the pre-IMS VRT block) being touched with a cotton bud on a specularly congruent location, either in synchrony or asynchrony with respect to the touch delivered on the participants' face (Figure 3B). After each IMS block, a new VRT block was run (post-IMS VRT), where the Outgroup face was the face seen during the previous IMS block (i.e., Outgroup Model 2; Figure 3C). The session ended by collecting participants' level of agreement with the randomly presented items of the questionnaire using a Visual Analog Scale (Figure 3D).

In each session we used different faces (i.e., for the second session we used Ingroup Model 3 and Outgroup Model 3 for the pre-IMS VRT; Outgroup Model 4 during the IMS and Ingroup Model 4 and Outgroup Model 4 for the post-IMS VRT). Thus, a different face was used in each VRT block and the assignment of each face to the different experimental blocks was counterbalanced across participants to control for any confounds related to idiosyncratic features of the models.

As in Experiment 1, accuracy scores were computed for each Face for each *bilateral* touches condition (i.e., Touch and No-Touch fingers' trajectories) separately, by measuring the percentage of correctly reported bilateral touches delivered on the participant's face, while viewing the Ingroup face being touched (Ingroup_Touch condition), the Outgroup face being touched (Outgroup_Touch condition), the Ingroup face being just approached (Ingroup_No-Touch condition) and the Outgroup face being just approached (Outgroup_No-Touch condition). These accuracy scores were calculated before and after each type of IMS (synchronous and asynchronous). Furthermore, in line with Experiment 1 we computed an index of the VRT effect as the difference in accuracy in detecting bilateral tactile stimulation in the Touch and No-touch fingers' trajectories, for each experimental condition (see Table 3). Finally, for each face we computed an index of the *change* in the VRT effect, by subtracting the previously computed VRT effect pre-IMS, from the VRT effect post-IMS (i.e., post- minus pre-IMS VRT effect), for each IMS condition (synchronous and asynchronous).

**Table 3:** **Mean scores (±standard error of the means indicated in brackets in italic font) for each face (Ingroup and Outgroup), for each Fingers' Trajectory (Touch and No-Touch), for each timing of the VRT task (pre- and post-IMS) and for each type of IMS (synchronous and asynchronous visuo-tactile stimulation between the face of the model and the face of the participant)**.

		**Stimulation**							
		***Synchronous***				***Asynchronous***			
		**Pre-IMS**		**Post-IMS**		**Pre-IMS**		**Post-IMS**	
		**M_ACC_**	**VRT effect**	**M_ACC_**	**VRT effect**	**M_ACC_**	**VRT effect**	**M_ACC_**	**VRT effect**
Ingroup	Touch	77% (3%)	12% (4%)	81% (3%)	8% (4%)	79% (3%)	13% (3%)	75% (3%)	10% (4%)
	No-Touch	65% (4%)		73% (4%)		66% (4%)		65% (4%)	
Outgroup	Touch	76% (3%)	3% (4%)	74% (4%)	1% (5%)	83% (3%)	8% (4%)	70% (3%)	2% (3%)
	No-Touch	73% (4%)		73% (4%)		75% (4%)		68% (4%)	

For each condition the VRT effect is calculated as the difference between Touch and No-Touch Fingers' Trajectories.

### Results

To show that synchronous IMS was able to induce the enfacement illusion, we compared the answers to each of the 12 statements of the questionnaire for the synchronous and asynchronous conditions using non-parametrical statistical tests (Wilcoxon Signed Ranks Test) (alpha level at 0.05). As expected (Tajadura-Jiménez et al., 2012b), synchronous stimulation produced higher scores compared to asynchronous stimulation across different dimensions (Table 2), such as identification with the other's face (Q1: *z* = 3.614; *p* = 0.000; Q2: *z* = 2.684; *p* = 0.007; Q3: *z* = 4.054; *p* = 0.000), changes in the perceived physical similarity (Q4: *z* = 3.409; *p* = 0.001; Q5: *z* = 2.467; *p* = 0.014) and in touch referral (Q11: *z* = 3.193; *p* = 0.001; Q12: *z* = 4.182; *p* = 0.000). Thus, synchronous IMS consistently produced significant changes in the way participants experienced the other face, suggesting that the other's face was embodied into self-face representation, although it did not belong to one's own ethnic group.

To test whether embodiment of an outgroup face into self-representation through synchronous IMS alters the racial bias in the VRT effect, we compared the strength of the VRT for ingroup and outgroup faces before and after IMS. To this aim, we first computed an index of VRT as the difference between detection of bilateral tactile stimuli in the Touch and No-Touch condition, as for Experiment 1 (see Table 3 for all mean accuracy scores and their standard errors for each experimental condition).

High values indicate stronger VRT effect, while low or negative values indicate poor or no VRT effect. We then ran a 2 × 2 × 2 ANCOVA on VRT indices with the within-subjects factors of Face (Ingroup and Outgroup), Stimulation (Synchronous and Asynchronous IMS) and Time (Pre- and Post-stimulation IMS), and with the IAT scores, as covariate, as in Experiment 1. The main effect of Face was significant [*F*_(1, 28)_ = 6.14, *p* < 0.05] because overall the VRT effect was bigger for Ingroup faces (*M* = 11%, s.e.m.= 2%) than for Outgroup faces (*M* = 4%, s.e.m. = 2%). The interaction Stimulation × IAT was also significant [*F*_(1, 28)_ = 4.88, *p* < 0.05], showing an increase of the VRT index as the IAT scores decrease, in the Synchronous [*r* = −0.47, *p* < 0.01], but not in the Asynchronous session [*r* = 0.08, *p* = 0.67]. Importantly, however, that interaction depends on the significant 4-way Stimulation × Time × Face × IAT interaction [*F*_(1, 28)_ = 5.92, *p* < 0.05], suggesting that synchronous and asynchronous IMS differently modulate the VRT effect for ingroup and outgroup faces depending on pre-existing individual differences in implicit racial bias.

**Figure 4:**
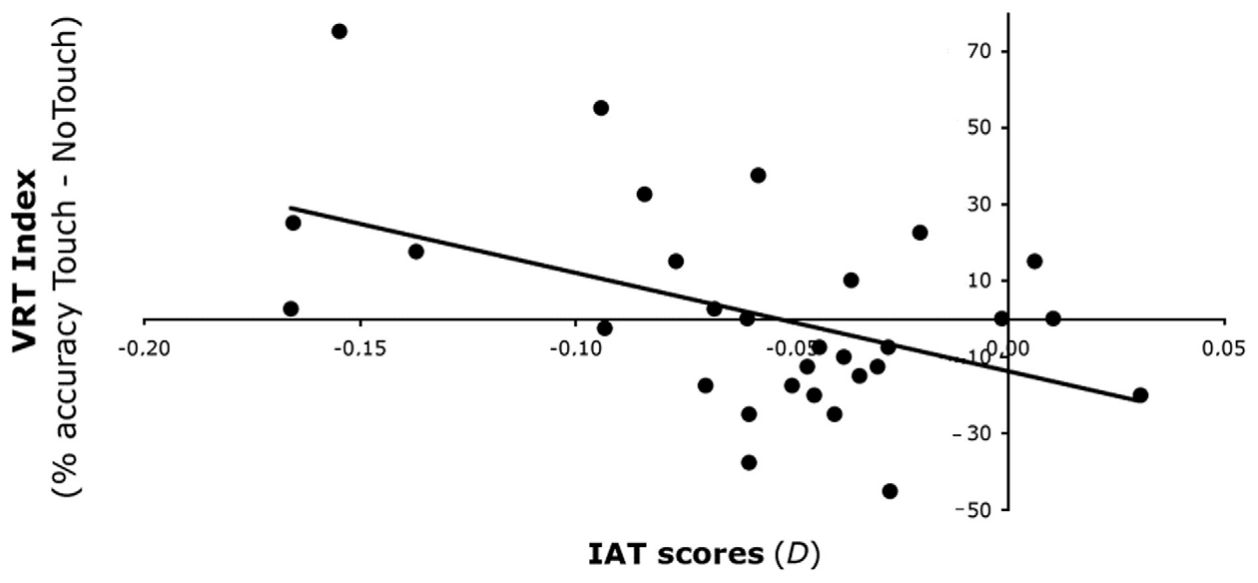
**Experiment 2 results.** Relationship between VRT effect for Outgroup faces and IAT scores after Synchronous IMS. After Synchronous IMS IAT scores (on the *x*-axis) predicted the increase of the VRT effect for Outgroup faces (on the *y*-axis)—expressed as the difference in detection of bilateral tactile stimulation in the Touch and No-Touch fingers' trajectory conditions.

To investigate the source of this significant interaction, we first split the analysis in two separate ANCOVAs for Synchronous and Asynchronous IMS, each with the within subjects factors of Face (Ingroup and Outgroup) and Time (Pre- and Post-stimulation), and with the IAT scores, as covariate. Whereas for the Asynchronous IMS no significant main effects nor interactions were found (all *p* > 0.24), for the Synchronous IMS a significant main effect of Face was found [*F*_(1, 28)_ = 5.14, *p* < 0.05], with higher VRT indices for Ingroup (*M* = 10%, s.e.m. = 3%) than for Outgroup faces (*M* = 2%, s.e.m. = 3%). Importantly, however, also a significant interaction Face × Time × IAT was obtained for the Synchronous IMS [*F*_(1, 28)_ = 4.27, *p* < 0.05]. To investigate how IMS interacts with the VRT effect for outgroup faces in relation to levels of implicit bias, we correlated the IAT scores with the VRT effects for the Outgroup face obtained after Synchronous stimulation. A significantly negative correlation [*r* = −0.475, *p* < 0.01] between IAT scores and the VRT effect after Synchronous stimulation showed that the more negative the attitude toward Outgroup faces—i.e., lower IAT scores—the greater the enhancement of tactile perception when viewing touch on Outgroup faces (Figure 4). As a control, the same analysis was conducted for the Asynchronous stimulation, and in that case, no significant correlation was found between IAT scores and the VRT effect [*r* = 0.183, *p* = 0.33]. In addition, the VRT effect for Ingroup faces was not affected by the IMS in relation with individuals' racial bias (all *p* > 0.22).

Finally, we computed an index of the *change* in the VRT effect (i.e., post- minus pre-IMS VRT effect) and correlated this with the IAT scores. A marginally significant negative correlation [*r* = −0.33, *p* = 0.07] suggested that participants with more positive attitudes toward racial outgroup members maintained constant their ability to remap touch seen on a black face; conversely, those prejudiced people whose initial somatosensory resonance was lacking, after synchronous IMS showed an increased ability to remap touch seen on an outgroup member.

## Discussion

The ability to remap onto one's body what we observe on the body of others is considered essential for social cognition (Keysers and Gazzola, 2009). However, such remapping is more automatic and effective when people interact with ingroup members (Serino et al., 2009; Avenanti et al., 2010). Here we show that individual differences in racial bias constrain the implicit resonance with others in the domain of somatosensory mapping (Experiment 1) and, moreover, that differences in somatosensory mapping while observing outgroup members can be altered as a result of changes in self-representation (Experiment 2).

In Experiment 1, the efficiency with which individuals remap tactile events observed on outgroup faces depended on the individuals' pre-existing implicit racial bias. Some theorists have suggested that when interacting with outgroup members, prejudiced individuals tend to focus more on the race stereotypes, categorizing the other-race face at the group level, ignoring individual differences (Ferguson et al., 2001; Lebrecht et al., 2009). The lack of VRT effect for the outgroup face as the implicit prejudice increases, can be related to this tendency to process a racial outgroup face at a category level, thus making trivial any attempt to remap observed physical states when they do not refer to an individualized single person.

Importantly, however, in Experiment 2, visual remapping for outgroup members was altered by changing one's self-representation. In particular, shared synchronous multisensory experiences with outgroup members—as those that induce the enfacement illusion (Tsakiris, 2008; Sforza et al., 2010)—enhanced the VRT effect for outgroup faces, but this effect was critically modulated by individual levels of implicit bias. Participants with strong negative biases against the outgroup members, indeed, showed higher changes in the VRT effect after IMS, while the absence of such modulation in participants without strong negative biases might reflect a ceiling effect in their ability to visually remap tactile events observed in outgroup faces.

The gradual incorporation of the other's facial features into the mental representation of one's own face, occurring during the synchronous IMS, might induce the outgroup face to be processed at an individual-, rather than at a categorical-level (Levin, 1996, 2000; Ferguson et al., 2001). This eventually results in an increased tendency to remap the sensory experience seen on the outgroup face onto ones' somatosensory system. This effect is in line with the changes induced on body image by illusory body-ownership, as people report increased perceived physical similarity between the newly attributed body-parts and their own bodies (Longo et al., 2009).

Other studies have demonstrated effective ways of altering implicit racial bias. Inzlicht and colleagues (Inzlicht et al., 2012) showed that the behavioral mimicry of an individual from a racial outgroup reduced implicit racial prejudice toward that outgroup, and suggested that mimicry reduced implicit prejudice by increasing self-other overlap, thus enhancing neural resonance with the racial outgroup. Maister and colleagues showed that induced changes in body-ownership for body parts of different skin color than one's own body also reduced implicit bias (Maister et al., 2013). Here we show how shared multisensory stimulation between two individuals belonging to different ethnic groups can change the extent of somatosensory resonance as a function of individual differences in implicit bias.

The present results suggest a bidirectional link between the automatic mechanisms of sensorimotor resonance, such as the VRT effect—that involves low-level multisensory integration processes—and higher-level physical and conceptual representations of self and others, such as ethnic membership. On the one hand, we show that the former are constrained by the latter, such as self-other, ingroup-outgroup categorizations weight the extent to which others' sensory states are remapped onto one's own somatosensory system. On the other hand, however, low-level multisensory mechanisms, such as IMS, can blur the boundaries between self-other, ingroup-outgroup categories, thus in turn increasing sensorimotor resonance between self and others.

We speculated that during the 2-min of synchronous stroking, one might come to bind the observed touch and the felt touch as a result of the multisensory mechanism of intersensory bias (Stein and Meredith, 1993), whereby the more compelling sensory information, i.e., the tactile stimulation received on one's own face, biases the judgment about the other sensory information, i.e., the visual context where the tactile stimulation occurs. Therefore, intersensory bias induces a touch referral mechanism, whereby the seen touch (from one reference frame, i.e., the other's face) comes to be associated to the felt touch (arising from a different reference frame, i.e., one's own face), generating a sense of identification with the seen face (Tsakiris, 2008; Paladino et al., 2010; Sforza et al., 2010). Importantly a top-down influence from high-order mechanisms—such as ingroup-outgroup categorizations—defines the initial perceptual distance between the two reference frames, as being progressively larger for racial outgroup members as individual pre-existing implicit racial bias increases. Our results suggest that such individual differences also determine the effectiveness of IMS in blurring the ingroup-outgroup boundaries.

In conclusion, whereas previous studies contributed to highlight the multisensory basis of social cognition (Avenanti et al., 2010; Inzlicht et al., 2012; Maister et al., 2013), this is one of the first experimental investigations that uncovers the potential multisensory mechanisms that through a continuous interaction between low-level perceptual processes and high-level representations, *change* those factors that shape interpersonal relationships.

## Author contributions

Manos Tsakiris and Andrea Serino developed the study concept. All authors contributed to the study design. Testing and data collection were performed by Chiara Fini and Flavia Cardini. Flavia Cardini performed the data analysis and interpretation. Flavia Cardini drafted the manuscript, and Manos Tsakiris and Andrea Serino provided critical revisions. Ana Tajadura-Jiménez revised the manuscript. All authors approved the final version of the paper for submission.

### Conflict of interest statement

The authors declare that the research was conducted in the absence of any commercial or financial relationships that could be construed as a potential conflict of interest.
